# Computer-aided diagnosis of prostate cancer based on deep neural networks from multi-parametric magnetic resonance imaging

**DOI:** 10.3389/fphys.2022.918381

**Published:** 2022-08-29

**Authors:** Zhenglin Yi, Zhenyu Ou, Jiao Hu, Dongxu Qiu, Chao Quan, Belaydi Othmane, Yongjie Wang, Longxiang Wu

**Affiliations:** ^1^ Department of Urology, Xiangya Hospital, Central South University, Changsha, China; ^2^ National Clinical Research Center for Geriatric Disorders, Xiangya Hospital, Central South University, Changsha, China; ^3^ Department of Burns and Plastic Surgery, Xiangya Hospital, Central South University, Changsha, China

**Keywords:** deep neural networks (DNN), computer-aided diagnosis (CAD), prostate cancer localization, prostate cancer classification, multi-parametric magnetic resonance imaging (MP-MRI)

## Abstract

**Objectives:** To evaluate a new deep neural network (DNN)–based computer-aided diagnosis (CAD) method, namely, a prostate cancer localization network and an integrated multi-modal classification network, to automatically localize prostate cancer on multi-parametric magnetic resonance imaging (mp-MRI) and classify prostate cancer and non-cancerous tissues.

**Materials and methods:** The PROSTAREx database consists of a “training set” (330 suspected lesions from 204 cases) and a “test set” (208 suspected lesions from 104 cases). Sequences include T2-weighted, diffusion-weighted, Ktrans, and apparent diffusion coefficient (ADC) images. For the task of abnormal localization, inspired by V-net, we designed a prostate cancer localization network with mp-MRI data as input to achieve automatic localization of prostate cancer. Combining the concepts of multi-modal learning and ensemble learning, the integrated multi-modal classification network is based on the combination of mp-MRI data as input to distinguish prostate cancer from non-cancerous tissues through a series of operations such as convolution and pooling. The performance of each network in predicting prostate cancer was examined using the receiver operating curve (ROC), and the area under the ROC curve (AUC), sensitivity (TPR), specificity (TNR), accuracy, and Dice similarity coefficient (DSC) were calculated.

**Results:** The prostate cancer localization network exhibited excellent performance in localizing prostate cancer, with an average error of only 1.64 mm compared to the labeled results, an error of about 6%. On the test dataset, the network had a sensitivity of 0.92, specificity of 0.90, PPV of 0.91, NPV of 0.93, and DSC of 0.84. Compared with multi-modal classification networks, the performance of single-modal classification networks is slightly inadequate. The integrated multi-modal classification network performed best in classifying prostate cancer and non-cancerous tissues with a TPR of 0.95, TNR of 0.82, F1-Score of 0.8920, AUC of 0.912, and accuracy of 0.885, which fully confirmed the feasibility of the ensemble learning approach.

**Conclusion:** The proposed DNN-based prostate cancer localization network and integrated multi-modal classification network yielded high performance in experiments, demonstrating that the prostate cancer localization network and integrated multi-modal classification network can be used for computer-aided diagnosis (CAD) of prostate cancer localization and classification.

## 1 Introduction

Prostate cancer is the most common malignant tumor of the male genitourinary system and has become the second most common malignant tumor in men worldwide, second only to lung cancer (Sung et al., 2021). Image information is of great significance for the diagnosis of prostate cancer. Transrectal prostate color Doppler ultrasound can be used as a screening tool for prostate cancer. Magnetic resonance examination is widely used to evaluate prostate cancer, and pathological examination and Gleason score are an important basis for prostate grading (Litwin and Tan, 2017). In the clinical diagnosis of prostate cancer, a radiologist is required to separate the prostate tissue from the surrounding tissues and organs in the prostate MRI. The meaningful information extracted by this segmentation process includes shape, the relative position of organs, volume, and abnormal signals. Because the area of ​​prostate tissue in MRI is small, less valid information is available, and the size, shape, and location of prostate tissue vary from patient to patient. Precise localization of the prostate and identification of prostate cancer remains difficult for radiologists.

In recent years, deep learning technology has developed rapidly in the medical field, which can extract features from image data in a supervised or unsupervised manner for image classification or segmentation. Deep neural network (DNN) is an artificial neural network that imitates the function of human neurons and can perform tasks such as classification (Ciresan et al., 2012), image segmentation (Quan et al., 2021), and entity reconstruction (Nguyen et al., 2020). It has a stronger expressive ability and can fit almost any function, but it also has problems such as many network parameters, a large training amount, and difficulty in training. The specific structure of DNN is shown in Supplementary Figure S1. The use of DNNs is growing exponentially, and researchers have used DNNs to correctly classify a large number of different classes of images (Deng et al., 2009). One of the main uses of DNN in medicine is to aid in the diagnosis of certain types of cancer, which are often identified clinically by skilled radiologists from medical images. Cancer detection methods based on artificial intelligence and MRI are widely used in daily clinical diagnosis, which has achieved higher diagnostic success rates than experienced radiologists. The study shows that the success rate of lung cancer detection and breast cancer detection using DNN is significantly better than the results of manual detection by radiologists (Becker et al., 2017; Coudray et al., 2018). In addition to the use of DNN on MRI, other studies have shown that the use of DNN can help determine the accuracy of results from transrectal biopsies for the diagnosis of prostate cancer (Takeuchi et al., 2019).

Therefore, DNN-based automatic localization of prostate MRI and prostate cancer diagnosis is a study with good clinical application prospects, which can assist radiologists in better diagnosis of prostate cancer. We propose a computer-aided diagnosis (CAD) method for localization and classification of prostate cancer based on DNN and mp-MRI, called prostate cancer localization network and integrated multi-modal classification network, aiming to improve the efficiency of radiologists. First, we used MR images provided by public prostate cancer databases and preprocessed them for training localization and classification models (Armato et al., 2018). The previously defined metric algorithm was then fully evaluated using a test set and a validation set. Finally, the output contains the image locations of possible malignancies and the likelihood of detecting prostate cancer based on the patient’s multi-parametric MRI.

## 2 Materials and methods

The goal of this study was to propose a new diagnostic assistant technology for prostate cancer, which uses multiple prostate MRIs of each patient as the input of the localization and classification model, and the output is the specific location of the localization and the classification of benign and malignant tissue, aiming to identify potential tumors. It is worth stating that our proposed localization network and classification network are both studied independently.

### 2.1 Data set

The PROSTATEx public database used in this study is part of the SPIE-AAPM-NCI Prostate MR Classification Challenge, which aims to advance the diagnostic classification of prostate cancer by analyzing prostate MRI (Armato et al., 2018). The database, collected by Radboud University Medical Centre (Radboudumc), covers more than 300,000 prostate MRIs from 346 patients, including T2-weighted image (T2WI), proton density–weighted image (PdWI), dynamic contrast enhancement (DCE), and diffusion-weighted (DW) images. Each patient has a Ktrans image, one or more DW images, and one or more T2 images.

In the DICOM file of the PROSTATEx dataset, the header information consists of the acquisition information of the image with the basic information of the case. Among them, the image acquisition information includes acquisition time, size, repetition time, pixel spacing, image position, and orientation. The dataset provides the coordinates of one or more points of interest (POIs) and information on the prostate area. Based on human experience, the characteristics of prostate lesions vary from region to region. Four prostate zones are associated with the POI provided: the peripheral zone (PZ), the transitional zone (TZ), the anterior fibromuscular stroma (AS), and the seminal vesicle (SV). The details of the dataset are shown in Table 1. The specific image classification of the database is shown in Table 2.

**TABLE 1:** Details of PROSTATEx dataset.

Category	PZ	TZ	As	SV	Total
Training set	191	82	55	2	330
Test set	113	59	34	2	208

AS, anterior fibromuscular stroma; PZ, peripheral zone; SV, seminal vesicle; TZ, transitional zone.

**TABLE 2:** PROSTATEx database image classification, including Ktrans, ADC, and t2-weighted images.

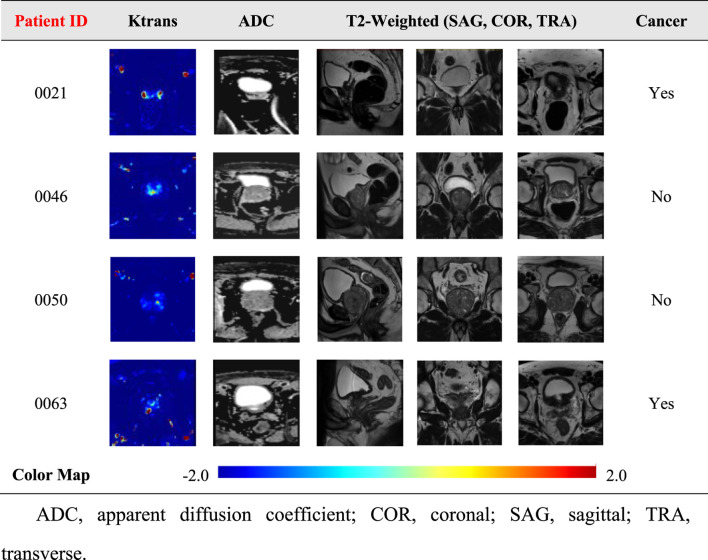

The training set of the PROSTAREx dataset contains 204 cases with 330 suspicious cancer lesions, of which 76 are gold standard “True” lesions, and the remaining 254 are gold standard “False” lesions. The test set contains 104 cases and 208 suspicious lesions to be diagnosed.

### 2.2 Data preprocessing

We observed the PROSTATEx dataset using ITK-SNAP and concluded that there are three problems in MR image preprocessing of the prostate: 1) abnormal data acquisition, such as missing sequences and different acquisition order; 2) different image resolution and gray value distribution among sequences; 3) insufficient sample size, containing only 330 training samples, and the network training is prone to overfitting.

**FIGURE 1:**
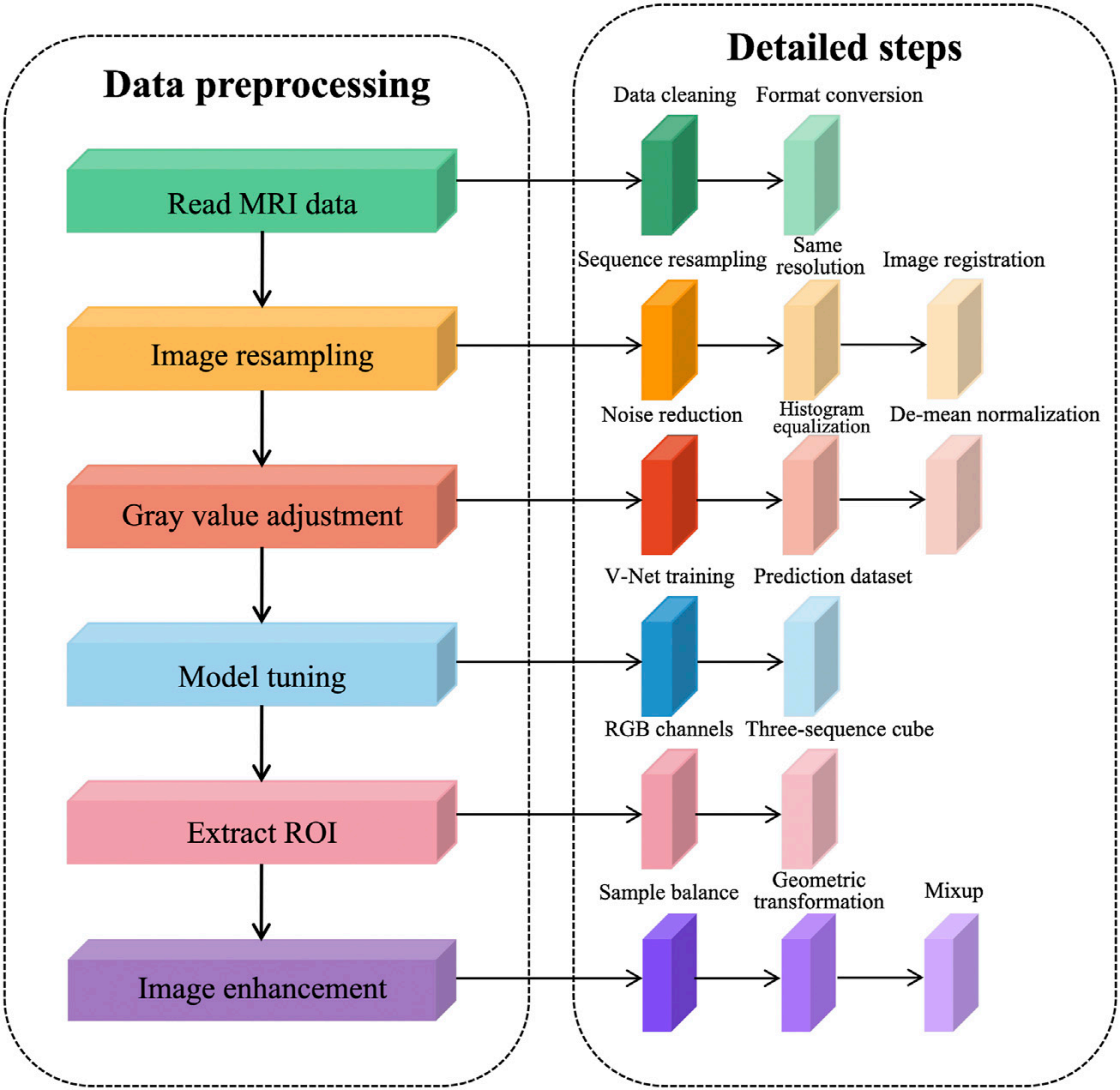
Prostate MRI data preprocessing steps.

To solve the problems existing in the dataset and further reduce the redundant information of network learning, the following preprocessing steps are proposed in this article, as shown in Figure 1. The prostate MRI data are first read so that the format is consistent between sequences, and then image resampling is performed so that the pixel spacing is consistent from case to case and from sequence to sequence. Next, the grayscale values are adjusted to ensure a consistent histogram distribution for each sequence. To further reduce redundant information, prostate tissue is extracted to reduce the learning of background information by the network. After generating a region of interest (ROI) that matches the network input structure, image enhancement is performed to expand the sample size.

We performed data cleaning on the training set and eliminated the cases with missing sequences. This decision reduced two cases, leaving 202 cases after elimination, each containing four sequences, namely, T2WI sequences, DWI sequences, Ktrans sequences, and ADC sequences. To expand the sample size, we used the common image enhancement methods of flip, pan, rotate, and zoom for multiple images of prostate MRI, and in addition, used the data enhancement method of Mixup to improve the linear expression between different samples.

**FIGURE 2:**
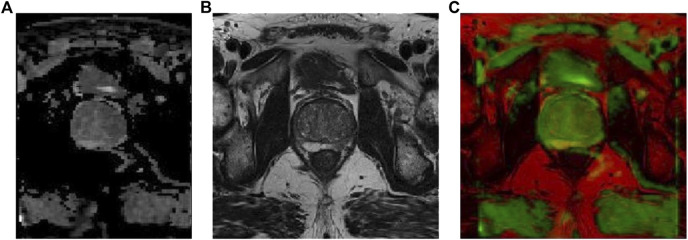
Prostate MR image alignment results, where **(A)** ADC image, **(B)** T2-weighted image, and **(C)** overlap map after image alignment.

The prostate alignment transformation used in this study is the B spline transformation. To make the alignment easier, two resolutions are used, first using a low resolution for the alignment and then a high resolution for the alignment. In performing the B spline transformation, a mutual information function with an increased penalty for rigidity is used as the optimization objective using an adaptive gradient descent algorithm. Finally, the rigid transform and B spline transformation were combined to obtain the final transform results. The results of prostate MR image alignment are shown in Figure 2.

### 2.3 Prostate cancer localization network structure

**FIGURE 3:**
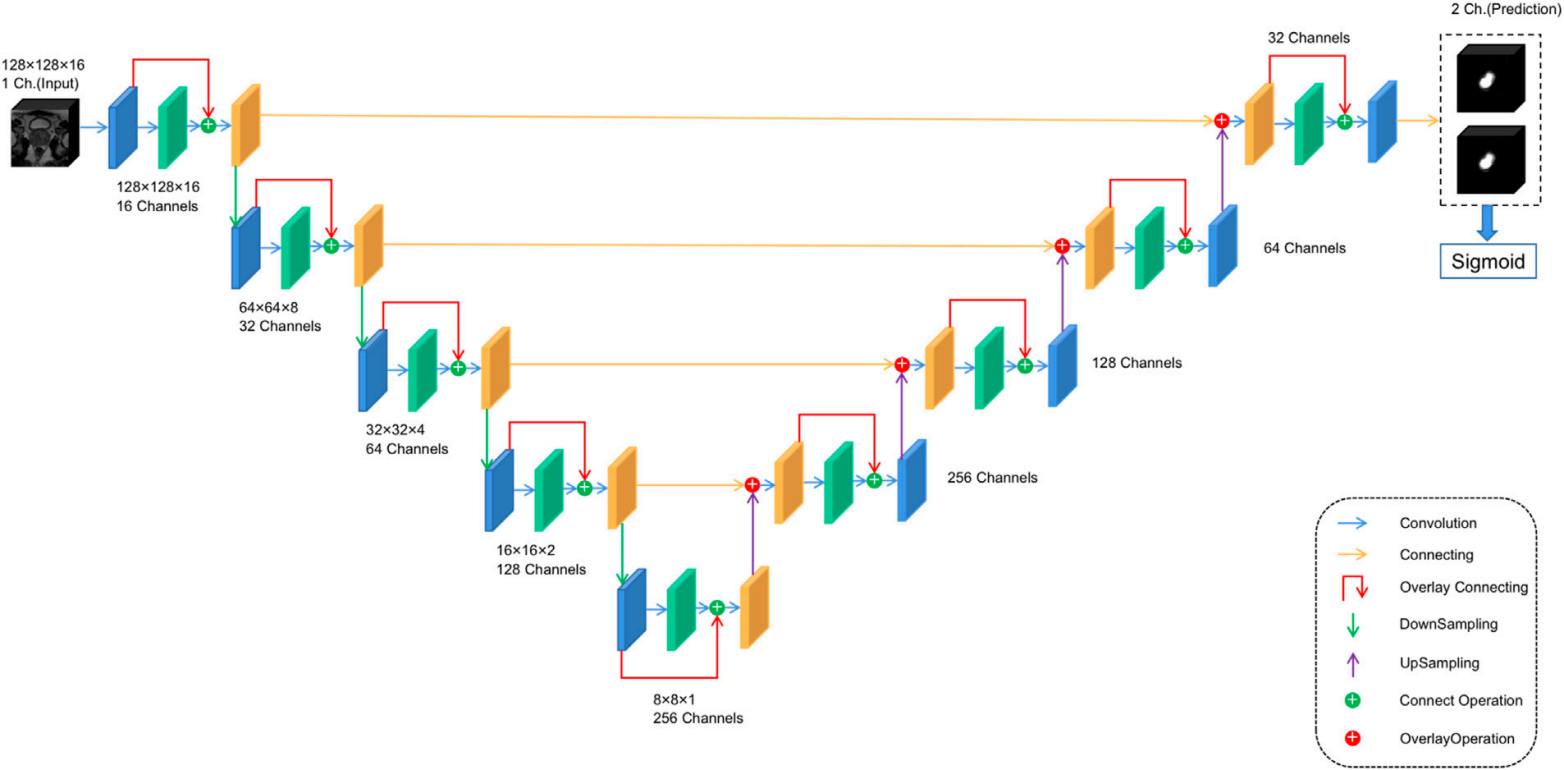
Network structure of the V-Net–based prostate cancer anomaly localization system.

For anomaly localization, previous research studies have created a 3D convolutional DNN specifically for medical image segmentation. The architecture used in this study is based on V-net (Milletari et al., 2016), a well-known image segmentation network for medical imaging. The main modification made in this study is the redefinition of the input and output tensors and activation functions. Both input and output tensors are of size (128,128,16,1), and Leaky ReLU is used as the activation function because the original PreLU activation function increases the risk of overtraining in small databases. At the same time, the output layer uses the sigmoid activation function, which can get the binary position of the tumor, and the output is reflected as the segmentation of the same position in the figure. The specific architecture is shown in Figure 3 to facilitate its repeatability.

### 2.4 Single-modal classification network structure

**FIGURE 4:**
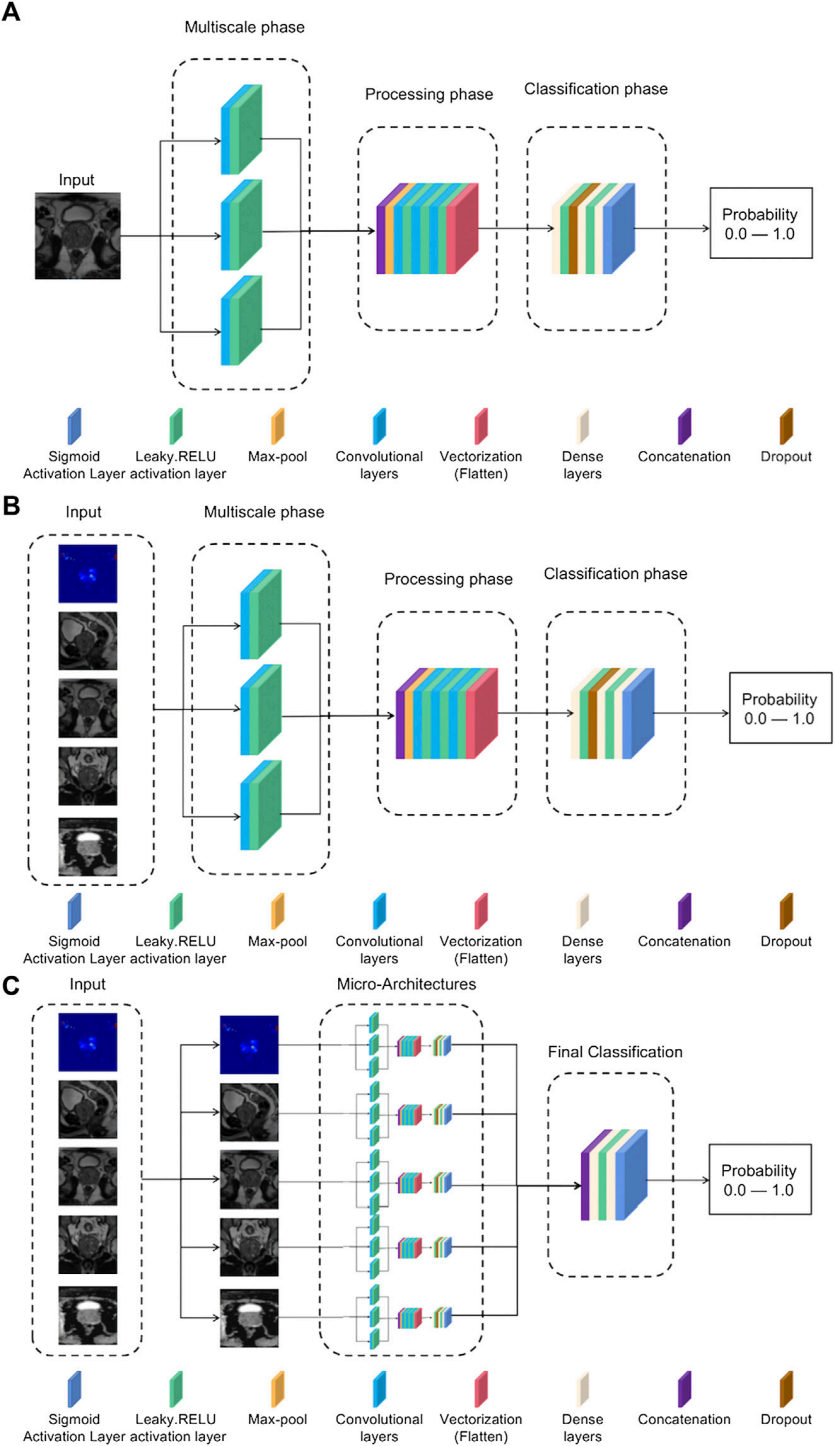
**(A)** Single-mode classification network structure. **(B)** Input tensor multi-modal classification network structure. **(C)** Integrated multi-modal classification network structure.

To address abnormal single-modal image classification, a lightweight architecture based on Inception-V3 and VGG-16 networks is proposed (Rueckauer et al., 2017). Typically, in this type of architecture, ReLU is chosen as the activation function (Dahl et al., 2013). However, to avoid problems such as gradient decay, LeakyReLU is still chosen as the activation function. The specific structure is shown in Figure 4A.

According to the order in the network structure, each process is introduced in turn:

1) Multiscale stage: Since tumors may be of different sizes or located in different locations, applying a series of multiscale 3D convolutions to the input data enables us to detect possible anomalies. This technique comes from the inception-v3 network, as it has been shown that multiscale filter banks can give good results on classification problems (Chollet and Ieee, 2017).
2) Processing stage: This stage starts from the max-pool layer, which allows obtaining the features of the maximum value. Convolutional filter banks are then used to obtain more complex features for further refinement of classification. This filter-based design is inspired by networks such as VGG-16 (Zhang et al., 2016). After this, a vectorized layer is used to unify all dimensions for dimensionality reduction.
3) Classification stage: The dense layer is used for classification. Experience has shown that the best training results are obtained using two dense layers with ten neurons.

### 2.5 Multi-modal classification network structure

Based on the single-modal classification network, we propose a multi-modal classification network structure, which tries to use multiple medical image data of patients and tries to combine the information of different attributes to achieve a better classification effect. This work proposes two different multi-modal classification network structures:

#### 2.5.1 Input tensor multi-modal classification network structure

The goal of the multi-modal classification network structure design is to have an accurate classification effect, and it can be trained using different modes of 3D volume channels to have multiple perspectives on the diagnosis of the same lesion location. The model uses five images of the same patient as input, for which it is necessary to preselect patients with more than five images available, reducing the training set. The rest of the neural network structure is the same as the single-modal classification structure in Figure 4A, but the input consists of five images each time instead of a single image. The specific network structure is shown in Figure 4B.

#### 2.5.2 Integrated multi-modal classification network structure

This model is the most complex in the article and is designed to use all the information previously obtained to generate a more accurate model. The network structure is based on the concept of multi-model ensemble learning (Xiao et al., 2018), which uses several lower-complexity classifiers to obtain a classifier with stronger performance. The model input uses all five types of images, but unlike the input tensor multi-modal classification structure, each type of image is now evaluated in its specific single-modal network structure, and the previously obtained weights are used to adjust the model parameters for best results. The outputs of these five sub-networks are processed in two convolutional layers, and the corresponding neurons use the leaky-RELU activation function and the Sigmoid activation function, respectively, to achieve the effect of binary classification. The specific structure is shown in Figure 4C.

### 2.6 Training parameters

Our model is implemented in python (version 3.8) and uses Tensorflow, Keras, OpenCV, and Cuda Toolkit as the backend DNN learning library.

We designed comparison experiments to select hyperparameters for training the classification network, including optimizers (ADAM, AdaGrad, and RMSProp), learning rate, epoch, and batch size. 1e^−4^ and 1e^−5^ learning rates were used to compare the performance of the algorithms in the comparison experiments. The model was applied to training with batches of sizes 4 and 8, while the corresponding epoch size grew from 50 to 200, increasing by 50 each time.

The parameters chosen for training the model are as follows.

1) Optimizer: The ADAM optimizer was used in this study (Zhang, 2018). The reason for choosing ADAM is that it combines the advantages of the two optimization algorithms, AdaGrad and RMSProp, and comprehensively considers the first-order moment estimation of the gradient (that is, the mean value of the gradient) and the second-order moment estimation (that is, the uncentered variance of the gradient) and calculates out the update step size. Parameter updates in ADAM are not affected by gradient scaling. Hyperparameters are well interpretable and usually require little or no tuning. At the same time, it can naturally realize the step size annealing process (automatically adjust the learning rate), which is very suitable for large-scale data and parameter scenarios such as medical image processing.
2) Batch size: Due to the small size of the database, the batch size was set to 4. This is a small-scale case and can lead to confusion in the direction of gradient descent.
3) Number of iterations: The number of iterations was set to 200, while retaining the weights of those excellent results in the validation set, thus, avoiding overfitting.
4) Learning rate: The learning rate was set to 1e^−5^, which is determined by the batch size.
5) Loss function: The loss function used in this study was focal loss, which is used to solve the problem of imbalance between positive and negative samples. The imbalance between positive and negative samples can cause the model training to fall into the local minimum of the loss function. Focal loss is used in medical image classification problems to reduce the weight of easy-to-classify samples so that the model can focus more on the hard-to-classify samples during training (Lin et al., 2020). The focal loss is calculated by multiplying the cross-entropy loss by the variable weights. Let p be the probability that the predicted sample is a positive sample ( p∈[0,1] ) and y denote the predicted outcome ( y∈{−1,1} ); then, the operation rules for the cross-entropy loss and focal loss of a sample are defined as follows:

$$CE\left(p,y\right)=\{\begin{array}{c} -\log\left(p\right),\text{if }y=1\\ -\log\left(1-p\right),\text{otherwise} \end{array}$$ (1)

$$FL\left(p,y\right)=\{\begin{array}{c} -{\left(1-p\right)}^{\gamma }\log\left(p\right),\text{if }y=1\\ -p^{\gamma }\log\left(1-p\right),\text{otherwise} \end{array}$$ (2)

When the sample is an easy-to-classify sample, i.e., the closer p is to 0 or 1, the smaller the calculated weight coefficient is, the smaller the proportion of the sample to the total loss, when constant; when the sample is a hard-to-classify sample, i.e., when p is close to 0.5, the larger the weight coefficient is, the larger the proportion of the sample to the total loss when *?* is constant. The focal loss used in this study makes *γ* = 2 to apply weights to the loss values of the hard and easy samples during the training process, making the model learning more focused on the hard-learned samples.

In the experiment, a five-fold cross-validation method was used to divide the 328 suspected lesions from the preprocessed PROSTATEx dataset into five folds according to the systematic classification to ensure that the distribution of data in each fold is consistent in terms of lesion area and benignity and malignancy, and also to avoid the problem of data leakage as much as possible. After five training sessions, the average AUC was taken as the final evaluation score.

### 2.7 Evaluation metrics

Network performance can be evaluated using metrics such as root mean square error (RMSE), true positive rate (TPR), true negative rate (TNR), F1-score and AUC, accuracy and confidence interval, Jaccard index, PPV, NPV, and DSC.

The RMSE is in the marked circle centroid, and the surface centroids obtained in the post-threshold prediction are calculated; RMSE is defined as follows:

$$\text{RMSE}\left(X,h\right)=\sqrt{\frac{1}{m}\sum_{i=1}^{m}{\left(h\left(x^{\left(i\right)}\right)-y^{\left(i\right)}\right)}^{2}}$$ (3)

where RMSE (X, *h*) is the loss function measured in the sample set using hypothesis *h*, and *h* is the prediction function of the system, also known as the hypothesis. *m* is the number of instances in the dataset, *x* ^*(i)*^ is a vector of all eigenvalues of the *i*th instance in the dataset, and *y* ^*(i)*^ is the expected output value.

TPR, TNR, PPV, and NPV are defined as follows:

$$\text{TPR}=\frac{\text{TP}}{\text{TP}+\text{FN}}=1-\text{FNR}$$ (4)

$$\text{TNR}=\frac{\text{TN}}{\text{TN}+\text{FP}}$$ (5)

$$\text{PPV}=\frac{\text{TP}}{\text{TP}+\text{FP}}$$ (6)

$$\text{NPV}=\frac{\text{TN}}{\text{TN}+\text{FN}}$$ (7)

F1-score is the harmonic value of the precision and recall evaluation indicators, the best value is 1, which is defined as follows:

$$F1-Score=2\cdot \frac{\text{TPR}\cdot \text{PPV}}{\text{TPR}+\text{PPV}}$$ (8)

AUC refers to the area under the ROC curve, which can be used to evaluate the classification quality of the classifier. The larger the value, the higher the quality of the classifier.

Accuracy is defined as follows:

$$\text{Accuracy}=\frac{TP+TN}{TP+TN+FP+FN}$$ (9)

The confidence in the accuracy is assessed using 95% confidence intervals, by which the range of the model’s accuracy for the overall sample can be estimated, and 95% confidence intervals are defined as follows:

$$\text{P}\left(\hat{\mu }-1.96\frac{\sigma }{\sqrt{\text{n}}}\leq \text{M}\leq \hat{\mu }+1.96\frac{\sigma }{\sqrt{\text{n}}}\right)\approx 0.95$$ (10)

where n represents the number of selected accuracies, $\hat{\mu }$ represents the mean of all accuracies, *σ* represents the standard deviation of all accuracies, and M represents the desired 95% confidence interval.

Jaccard index is used to compare the similarity and difference between finite sample sets. The larger the value of the Jaccard coefficient, the higher the sample similarity. Given two sets, A and B, the Jaccard coefficient is defined as the ratio of the size of the intersection of A and B to the size of the concurrent set of A and B. It is defined as follows:

$$J\left(A,B\right)=\frac{\left|A\cap B\right|}{\left|A\cup B\right|}=\frac{\left|A\cap B\right|}{\left|A\right|+\left|B\right|-\left|A\cap B\right|}$$ (11)

Dice similarity coefficient (DSC) is used to measure the similarity of two sets, the value range is (0,1), and the larger the value, the more similar the two sets, commonly used in calculating the similarity of the closed region, defined as follows:

$$DSC=\frac{2TP}{FP+2TP+FN}$$ (12)

## 3 Results

The performance of the selected different optimizers, learning rate, epoch, and batch size in the training network is shown in Supplementary Table S1. According to Supplementary Table S1, the Adam algorithm achieved the highest performance with a learning rate of 1e^−5^, 200 epochs, and 4 min-batches.

### 3.1 Prostate cancer localization network results

The results of the prostate cancer localization network are shown in Table 3. It presents experimental results from both quantitative and qualitative perspectives.

**TABLE 3:** Prediction results of prostate cancer localization network.

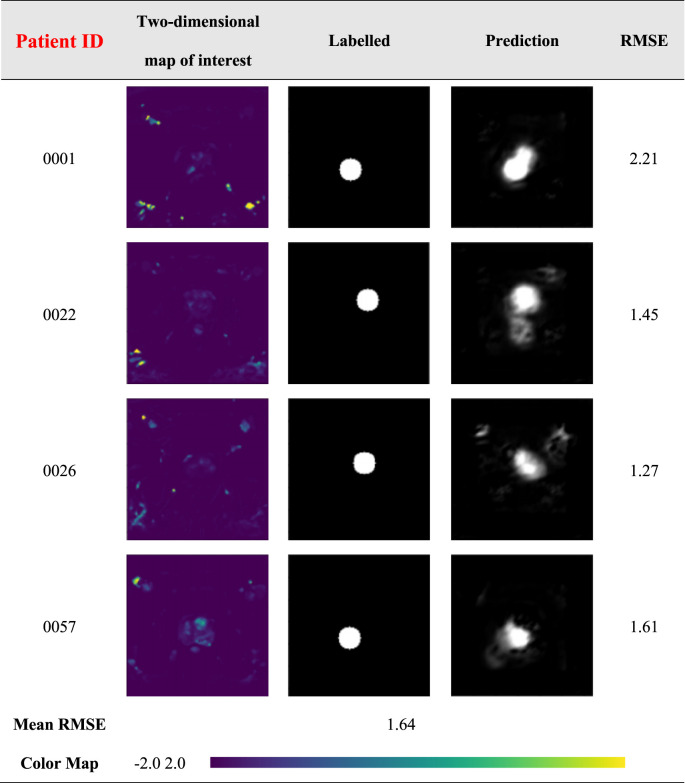

Table 3 shows the results for four different patients in the dataset. The first column shows the patient ID., The second column shows the 2D Ktrans map, represented by a “viridis” color band for better visualization. The third and fourth columns show the two-dimensional images of the prediction results of the artificial labeling and localization network after inputting Ktrans images, which are all grayscale images, and it can be observed that the prediction results are very close to the label image. Due to the small size of the prostate, it is, on average, 40 × 30 × 20 mm. Numerically, the error between the predicted results and the labeled results for the four patients was less than 3 mm, with an average error of only 1.64 mm, and the prediction results were only about 6% error compared to the normal prostate volume. Therefore, it can be considered that the prostate cancer localization network has excellent performance and accurate prediction results, and the results can be further improved by using a larger database or better data preprocessing in the future.

The first is the qualitative result, which visualizes the 2D portion of the 3D volume segmentation, and both the labeled images and the network predictions detect the presence of cancerous tissue. The second is the quantitative result, which expresses the root mean square error (RMSE) of each experimental image as the average RMSE in the database in millimeters while taking into account the resolution of the instrument and other issues. The use of viridis ribbons is intended to improve the readability of graphics for readers with common forms of color blindness and color vision deficiencies. Color graphics are also uniform in perception, both in regular form and when converted to black and white for printing. The performance of the prostate cancer localization network compared with previous classical segmentation methods is shown in Table 4.

**TABLE 4:** Performance of prostate cancer localization network compared with previous classical segmentation methods.

Model	Sensitivity	Specificity	Jaccard index	PPV	NPV	DSC
U-Net	0.80	0.83	0.79	0.76	0.80	0.74
U-Net++	0.82	0.84	0.82	0.81	0.83	0.75
DenseNet	0.86	0.88	0.87	0.85	0.89	0.81
FCN	0.85	0.89	0.86	0.90	0.89	0.82
SegNet	0.91	0.87	0.87	0.86	0.90	0.78
Our Method	**0.92**	**0.90**	**0.89**	**0.91**	**0.93**	**0.84**

DSC, dice similarity coefficient; NPV, negative predictive value; PPV, positive predictive value. Best performance values are in bold.

As shown in the table, the prostate cancer localization network proposed in this study has improved in each index compared with previous methods, with sensitivity, specificity, Jaccard index, PPV, NPV, and DSC of 0.92, 0.90, 0.89, 0.91, 0.93, and 0.84, respectively.

Once the model is trained using multi-modal datasets, the performance of the network can be quantitatively evaluated by volumetric or regional overlapping metrics, e.g., Dice scores, as stated in the study by Yang et al. (2022). The experimental data in Table 4 fully illustrate the interpretability of the model, which enhances its credibility and transparency of the model, and also facilitates future improvements of the model.

### 3.2 Single-modal classification network results

#### 3.2.1 Confusion matrix

**FIGURE 5:**
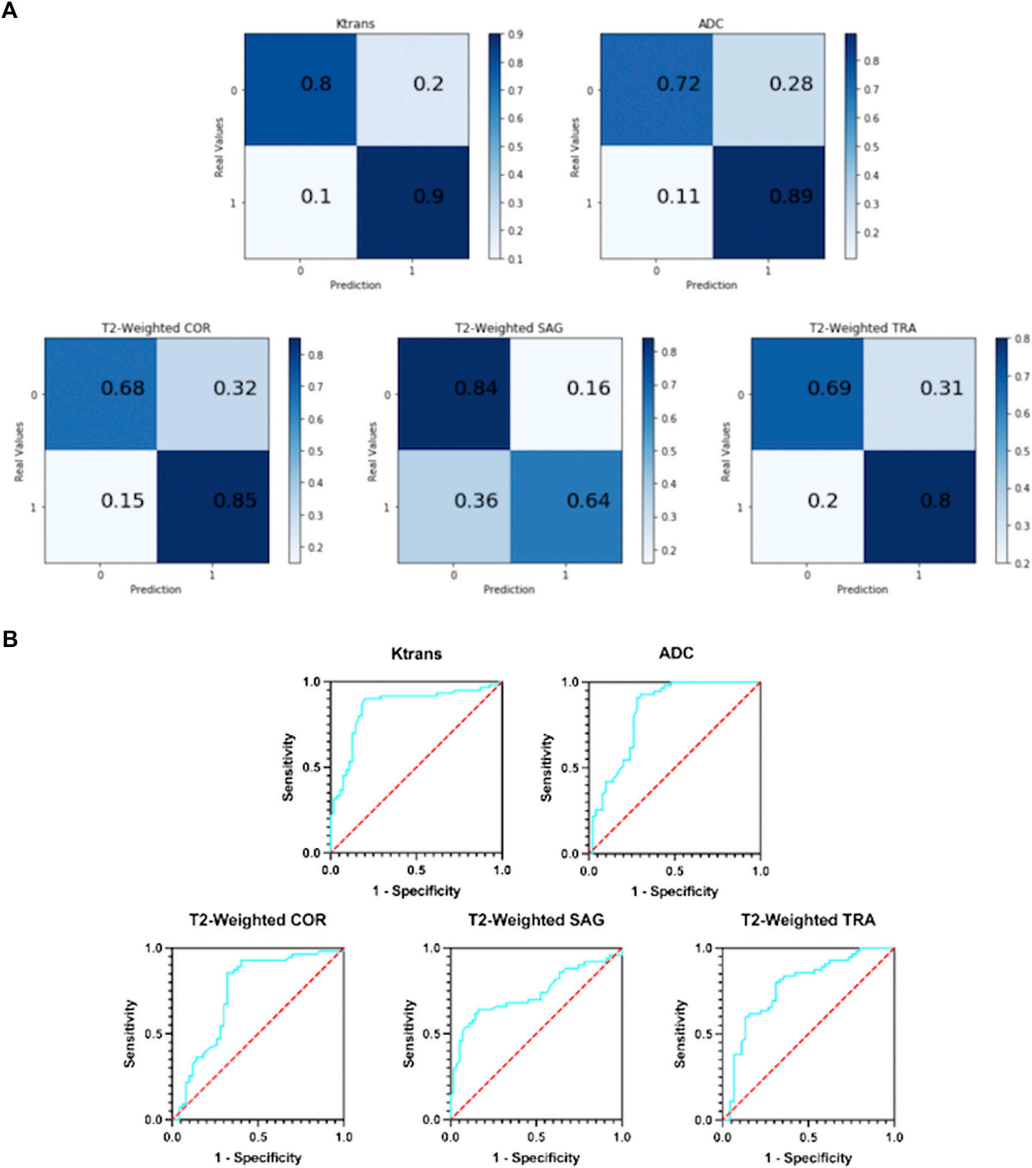
**(A)** Confusion matrix of five single-modal classification networks. **(B)** ROC curves of five single-mode classification networks.

Figure 5A shows the resulting confusion matrix for five different types of images as input to a single-modal classification architecture. According to Figure 5A, the Ktrans and ADC modal images perform best, and the true positive rate (TPR) and true negative rate (TNR) values are quite balanced, with an average of about 82% (85 and 80.5%, respectively).

In T2-weighted images, however, there was a large difference between the results of the three-parameter imaging. The COR image performed the best with an average of 77%, and its results were inferior to Ktrans and ADC. The mean values of SAG images and TRA images were 74 and 75%, respectively. They are not good indicators for detecting prostate cancer.

#### 3.2.2 ROC curve

Figure 5B shows the ROC curves of the results obtained with five different types of images as input to a single-modal classification network. The ROC curve graph is a curve reflecting the relationship between sensitivity and specificity. The *X*-axis of the abscissa is 1-specificity, also known as the false positive rate (FPR), and the closer the *X*-axis is to zero, the higher the accuracy; the *Y*-axis of the ordinate is called the sensitivity, also known as the true positive rate (TPR), and the larger the *Y*-axis, the better the accuracy. So, the closer the curve is to (0,1), the better its performance.

It can be seen from Figure 5B that the performance of the Ktrans and ADC modes is relatively better. Ktrans has a smoother curve and better response, while ADC has a more abrupt response. Compared with the former two, the curves obtained by T2-weighted COR, SAG, and TRA are less suitable for the detection of prostate cancer localization.

#### 3.2.3 Overall results

Supplementary Table S2 contains the comprehensive evaluation indicators of five single-mode classification networks, including TPR, TNR, F1-Score, AUC, and accuracy.

It can be observed that Ktrans performs the best, exceeding 0.85. ADC and COR performed slightly worse, stable at around 0.8. While SAG and TRA performed the worst, both less than 0.8. 0.8 was chosen as the threshold; based on this metric, the Ktrans modality was considered the most suitable input modality.

It can be observed that Ktrans and ADC still have good performance, reaching 85 and 83% of the area, respectively. The AUCs of the three modes of T2-weighted all fluctuate around 75%, which is not excellent.

The accuracy indicator selects 0.8 as the threshold. It was observed that among the five single-modal classification networks, Ktrans performed the best with an accuracy of 85%, followed by ADC with an accuracy of 81%.

### 3.3 Multi-modal classification network results

#### 3.3.1 Input tensor multi-modal classification network results

**FIGURE 6:**
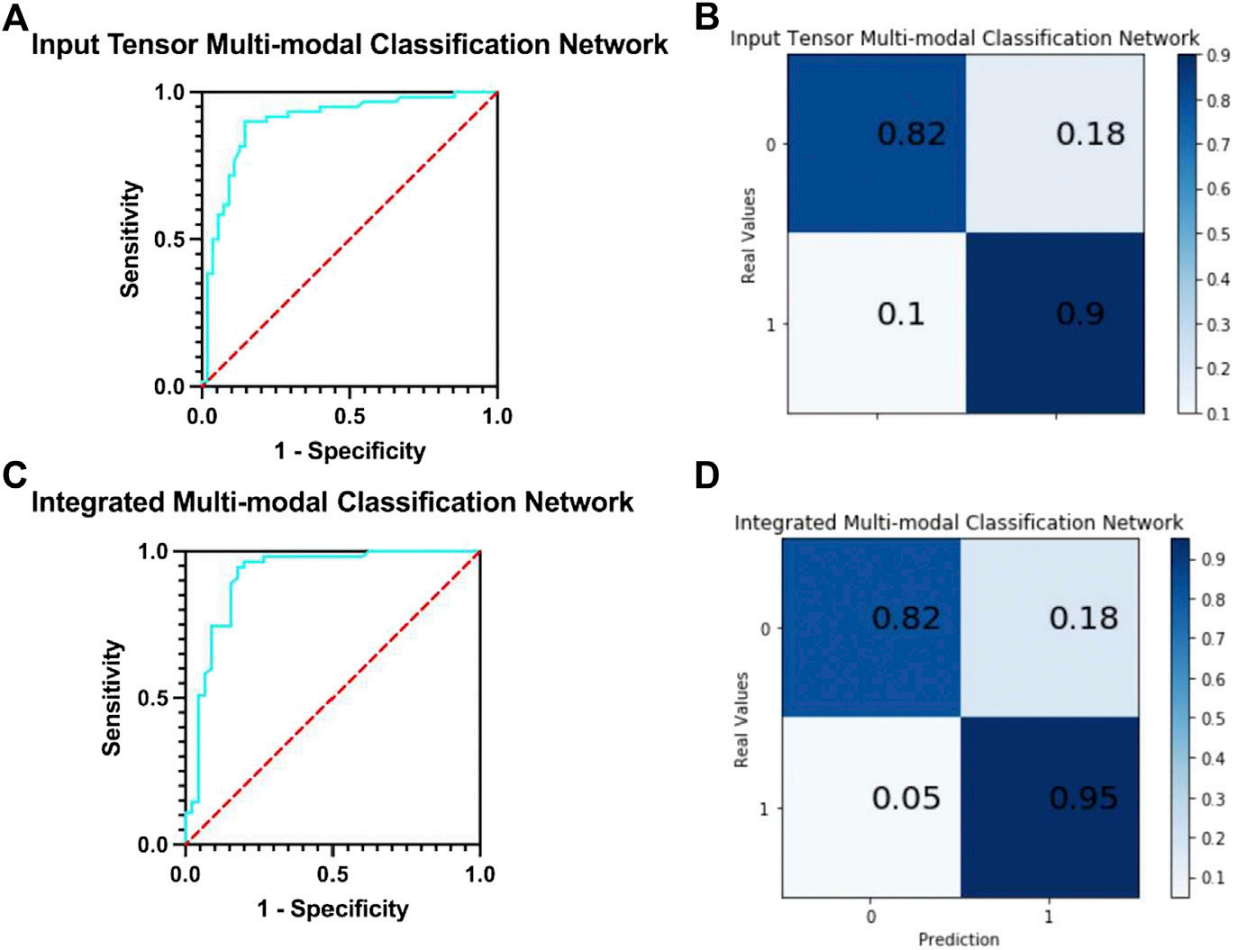
**(A)** ROC curve of input tensor multi-modal classification network. **(B)** Confusion matrix of input tensor multi-modal classification network. **(C)** ROC curve of integrated multi-modal classification network. **(D)** Confusion matrix of integrated multi-modal classification network.

In this network, five MRIs of the same patient with different modes are used as input. The following analyze and compare various indicators to judge whether the integration and fusion of the models can bring better performance. As shown in Figure 6A, the model obtained an AUC of 0.900, which is better than the highest value of 0.853 for the single-mode classification network. At the same time, it can be seen from the confusion matrix (Figure 6B) that the TPR and TNR values of the network are 90 and 82%, respectively, and the average value is 86%, which is slightly higher than Ktrans (average 85%), which is the best result obtained in single-mode networks and variants at present.

We compare the input tensor multi-modal classification network with five single-modal classification networks using detailed metrics in Supplementary Table S3. It can be observed that the input tensor multi-modal classification network outperforms the above single-modal classification network on almost all metrics, and although the lead may not be large, these small improvements play a role in the clinical detection and diagnosis of prostate cancer.

#### 3.3.2 Integrated multi-modal classification network results

The network integrates five single-mode classification models, in each of which images of the corresponding modality of the same patient are processed. The ROC curve of the network is shown in Figure 6C. The model obtained an AUC of 0.912, which is higher than all previous models proposed in this study and has the best classification performance with a 1.2% improvement over the results of the input tensor classification network.

The confusion matrix of the integrated multi-modal classification network is shown in Figure 6D; the values of TPR and TNR are 95 and 82%, respectively, which exceed the previous best values of 90 and 82% obtained by the input tensor multi-modal classification network. This shows that the integrated multi-modal classification network, through the integration of the single-modal classification model, is not only more robust and less coupled but also optimizes the results obtained by the single-modal classification network and the input tensor multi-modal classification network to a certain extent. To test this claim, Table 5 presents the data for the remaining indicators.

**TABLE 5:** Indicators of integrated multi-modal classification network, input tensor multi-modal classification network, and five single-modal classification networks.

Modality	TPR	TNR	F1-score	AUC	Accuracy
Integrated Multi-modal Classification Network	**0.95**	0.82	**0.8920**	**0.912**	**0.885**
Input Tensor Multi-modal Classification Network	0.90	0.82	0.8654	0.900	0.86
Ktrans	0.90	0.80	0.8571	0.853	0.85
ADC	0.89	0.72	0.8203	0.826	0.805
T2-Weighted COR	0.85	0.68	0.7834	0.741	0.765
T2-Weighted SAG	0.64	**0.84**	0.7636	0.735	0.74
T2-Weighted TRA	0.80	0.69	0.7583	0.775	0.745

ADC, apparent diffusion coefficient; AUC, area under curve; COR, coronal; TNR, true negative rate; TPR, true positive rate; SAG, sagittal; TRA, transverse. Best performance values are in bold.

Table 5 contains all the results for all the models in this study. The integrated multi-modal classification network has the optimal value for all the other indicators except TNR. For example, the prediction accuracy is improved by about 4% compared to the Ktrans single-modal classification network. Although the absolute value of the improved accuracy is not high, the higher the previous accuracy, the more significant the improvement obtained.

Meanwhile, we also conducted experiments on whether the reduction in the number of training samples would affect the classification performance of the model by setting the sample size to 50, 100, 150, and 200, respectively, and the network model was selected as the best-performing integrated multi-modal classification network in the abovementioned experiments, and the specific results are shown in Supplementary Table S4.

From Supplementary Table S4, it can be seen that the number of training samples increases from 50, 100 to 150, with the increase of training samples, the indexes have a large improvement, where the accuracy increases significantly by 20%, from 0.683 to 0.885. In the subsequent increase of training samples, from 150 to 200 to use all training samples, it can be seen that the accuracy is stable above 0.85, and the values of TPR, TNR, F1-score, and AUC indexes are stable around 0.92, 0.80, 0.86, and 0.88. It can be seen that the size of the training set has a certain influence on the performance of the classification system. With the increase of the training scale, the classification performance gradually improves, but after a certain scale, the classification performance does not change much and remains at a more stable value.

Table 6 shows the performance comparison of the integrated multi-modal classification network using some of the modalities for learning, divided into four groups for ablation experimental comparison.

**TABLE 6:** Effect of the number of modalities on model performance.

Modality	TPR	TNR	F1-score	AUC	Accuracy
Ktrans + ADC	0.91	0.80	0.8575	0.864	0.851
Ktrans + T2-Weighted	0.89	0.81	0.8424	0.859	0.834
ADC + T2-Weighted	0.87	0.81	0.8281	0.853	0.842
Ktrans + ADC + T2-Weighted	**0.95**	**0.82**	**0.8920**	**0.912**	**0.885**

AUC, area under curve; TNR, true negative rate; TPR, true positive rate. Best performance values are in bold.

Table 7 shows the comparison of the average accuracy 95% confidence intervals of the three models mentioned in the article and their different modalities. It can be observed that the average accuracy 95% confidence interval of the integrated multi-modal classification network has the least fluctuation of 0.004, which indicates that this model is more stable compared to other models.

**TABLE 7:** Comparison between different classification networks, stratified by accuracy and 95% confidence interval.

Model	Modality	Average accuracy, 95% confidence interval
Integrated Multi-modal Classification Network	-	**0.885** [0.881, 0.889]
Input Tensor Multi-modal Classification Network	-	0.86 [0.852, 0.868]
Single-modal Classification Network	Ktrans	0.85 [0.84, 0.86]
	ADC	0.805 [0.702, 0.818]
	T2-Weighted COR	0.765 [0.75, 0.78]
	T2-Weighted SAG	0.74 [0.721, 0.759]
	T2-Weighted TRA	0.745 [0.727, 0.763]

To evaluate the classification network proposed in this article, previous networks designed using the PROSTATEx dataset were selected for comparison, as shown in Table 8.

**TABLE 8:** Comparison of the classification model proposed in this article with the results of previous classification models.

Model	Author	AUC
Inception V3	Quan Chen	0.83
VGG-16	Quan Chen	0.81
XmasNet	Saifeng Liu	0.84
SVM	Jarrel C.Y. Seah	0.84
3D Convolutional Neural Networks	Alireza Mehrtash	0.80
Single-modal Classification Network	-	0.853
Input Tensor Multi-modal Classification Network	-	0.900
Integrated Multi-modal Classification Network	-	0.912

## 4 Discussion

Our method successfully achieves accurate segmentation of the prostate on magnetic resonance images, and experiments with the prostate cancer localization network obtained an average root mean square error of 1.64 mm, which is approximately less than 6% error compared to the normal size of the prostate. The error of 6% is an acceptable error range, which indicates that the localization network of the prostate proposed in this study possesses a good performance. Compared with the classical medical segmentation network U-Net, the method in this study has improved by 0.12 and 0.07 in sensitivity and specificity, respectively. In terms of the Jaccard index, the performance of DenseNet, FCN, and SegNet is respectable and slightly lower than the results of this study’s method by 1–3%. The prediction results of the prostate cancer localization network can be used as an evaluation index to assist radiologists in diagnosis so that doctors can locate prostate cancer more quickly and accurately.

Grand Challenges and the SPIE Medical Imaging Symposium launched an open competition in 2017 on prostate cancer prediction on magnetic resonance images to promote advances in prostate cancer detection algorithms (Litjens et al., 2014). Currently published research studies on deep learning–based prostate classification algorithms are mainly focused on PROSTATEx contestants published in *PROCEEDINGS OF SPIE*, where the use of convolutional neural networks is mostly based on the abovementioned VGG network modification. Chen et al. used a migration learning approach with Inception V3 and VGG-16, pre-trained on ImageNet, as the base network (Simonyan and Zisserman, 2014; Szegedy et al., 2016). In addition, because of the different number of positive and negative sample distributions in the cancer lesion regions, a network was trained on each region, and finally, the results of the different networks were weighted and averaged. The performance of the competition results on the PROSTATEx test set is AUC = 0.83 and AUC = 0.81, respectively (Chen et al., 2017). Liu et al. also built a new deep learning architecture, called XmasNet, based on VGG net, and obtained seven results by combining training between different sequences and calculating the weights of the seven models using a greedy algorithm, and the prediction results were taken as a weighted average, and the performance on the test set was reflected as AUC = 0.84 (Liu et al., 2017). Similar to their study, we first propose a single-modal classification network structure based on Inception-V3 and VGG-16 networks. Based on this, we further propose an input tensor multi-modal classification network structure. Combined with multi-modal ensemble learning, we propose an integrated multi-modal classification network structure. The multi-modal classification network combines the current emerging multi-modal learning and ensemble learning techniques to transfer the knowledge learned on the information-rich modality to the information-poor modality so that the learning of each modality can assist each other to achieve better classification results (Xiao et al., 2018). The integrated multi-modal classification network improved the AUC by 8.2 and 7.2% compared to Chen et al. and Liu et al.'s network, respectively, with an AUC of 0.912.

Mehrtash et al. designed a three-branch three-dimensional convolutional neural network to exploit the spatial information of the lesion and introduced regional information of the lesion location in the fully connected layer. The CNN architecture consists of three input streams: ADC map, maximum b-value from DWI, and Ktrans from DCE-MRI. Its model input is a 32*32*12 3D ROI centered on the lesion. The prediction result on the test set is AUC = 0.80 (Mehrtash et al., 2017). Unlike their design, our input stream also includes T2-weighted images, and conventional T2WI has a greater diagnostic value for prostate cancers occurring in the peripheral zone, where 70–80% of prostate cancers are clinically located (Lee et al., 2015; Israel et al., 2020). Therefore, the T2-weighted image is not only indispensable for unimodal classification networks but also has an active role in multi-modal fusion learning. Seah et al. concluded that the contrast and brightness of prostate MR images are important factors affecting the judgment of the benignity and malignancy of lesions, so they designed the auto windowing module, which can adjust the contrast and brightness of images adaptively according to the input data and reduce the steps of image preprocessing. In addition to this, additional information such as the patient’s age, the area, and the angle at which the lesion was located was used. Finally, by model integration, the network had an AUC = 0.84 on the test set (Seah et al., 2017). For the characteristics and problems of the PROSTATEx challenge dataset, we proposed image alignment, resampling, noise reduction, and normalization preprocessing methods in this study. To solve the problem of small sample data, we propose image panning, rotation, zooming, flipping, and Mixup image enhancement methods, and finally, achieve an AUC of 0.912.

As can be seen from Table 8, the AUCs of the integrated multi-modal classification network and input tensor multi-modal classification network models proposed in this study are both significantly better than the mentioned existing mainstream classification models, which are 0.912 and 0.900. Compared with the best-performing SVM and XmasNet, the integrated multi-modal classification network improves the AUC by 7.2%. Therefore, it can be proved that the proposed integrated multi-modal classification network has better classification performance.

The experimental results of our classification network show that the Ktrans modality in the single-modal classification network performs the best with an accuracy of 85%. Subsequently, by integrating and fusing different classifiers, the accuracy of the input tensor multi-modal classification model was improved to 86%. Finally, the best results are achieved in the integrated multi-modal classification model, with a small improvement of 2.5% and an accuracy of 88.5%. Therefore, we can conclude that the integration and fusion of models can lead to better performance, and the input tensor multi-modal classification network improves the performance by 1–2% compared to the single-modal classification network. On this basis, the performance of the integrated multi-modal classification network is improved by 2.5% compared to the input tensor multi-modal classification network. The successful integration of multiple models not only makes the new structure more robust and achieves the goal of low coupling but also proves that images can be combined in a decoupled manner because each single-modal classification model can be trained in a decoupled manner, and only the final network weights need to be adjusted. Huang et al. showed that the quality of the latent representation space directly determines the effectiveness of the multi-modal learning model, and the richer the variety of modalities, the more accurate the estimation of the representation space and the better the learning effect with sufficient training data (Huang et al., 2021). As can be seen from Table 6, the combination of Ktrans + ADC + T2-weighted with the highest number of modalities still achieves the best performance in all evaluation metrics, and the modal combination of Ktrans + ADC performs well in TPR, F1-Score, AUC, and accuracy, but not as well as Ktrans + T2-weighted and ADC + T2-weighted in TNR. This suggests that although T2-weighted images do not perform as well as Ktrans and ADC on single-modal classification networks, they have an active role in multi-modal fusion learning. Taking the assisted diagnosis of prostate cancer MRI as an example, multi-modal learning can aggregate information from multiple sources of data, make the representation learned by the model more complete, transfer the knowledge learned on the information-rich modality to the information-poor modality, and make the learning of each modality assist each other to achieve better classification results.

Both the prostate cancer localization network and the single-modal and multi-modal classification networks have achieved good results, but these models cannot be considered accurate enough to be used as a single diagnostic criterion. It is better suited as a support system or second opinion for radiologists, capable of detecting overlooked positive cases or speeding up the detection of possible positive cases.

Other publicly available prostate MRI datasets can be used in the future to optimize model training with the study of prostate cancer tissue contour segmentation, such as the PROMISE12 competition dataset, the main theme of which is prostate segmentation using T2WI sequences of the prostate. The data provided include 50 training samples and the corresponding prostate masks and 30 test samples. Also, in the future, when facing the multicenter prostate cancer MRI data fusion problem, it is necessary to consider the problem of certain disparity in imaging results due to scanner, parameters, and environment (Nan et al., 2022). In addition, it is possible to use a deep learning-based approach to construct scanner image invariant encoding based on the existing methods (Moyer et al., 2020). As for the interpretability of the model, in the next step, we add visual interpretation methods such as gradient interpretation method, GradCAM interpretation method, and RISE interpretation method to further solve the problem of opaque model details and achieve a “trustworthy” and “interpretable” diagnosis process.

Our study has some limitations. First, medical ethics requires that the effectiveness and safety of any new technology in the clinical application must be fully tested. Medical artificial intelligence alone has certain risks in judging diseases based on imaging data. The results of this study can only be used as a reference for radiologists’ diagnoses. Second, our research is purely based on mp-MRI and does not add other types of medical indicators as parameters to the design and training of the model, such as the patient’s age, weight, and PSA, to improve the generalization ability of the model. Third, in the diagnosis of prostate cancer, the DNN technology based on magnetic resonance examination is based on its database or public database and lacks external verification of a large sample size, which is also our future research direction. We look forward to developing new single-modal classification models in future work that achieve higher accuracy in the T2-weighted modality, thereby indirectly improving the performance of an integrated multi-modal classification network. Furthermore, we will cooperate with the Radiology Department of Xiangya Hospital to create our database and test our system in a real medical environment and consider inter-observer variability.

## 5 Conclusion

CAD of prostate cancer remains a challenging topic. In this article, we propose a localization and classification network for prostate cancer based on DNN and mp-MRI to assist radiologists in the diagnosis of such diseases. We constructed four different localization and classification networks, namely, prostate cancer localization network, single-modal classification network, input tensor multi-modal classification network, and integrated multi-modal classification network, and analyzed them in detail through experiments. The results show that the DNN-based prostate cancer localization network and integrated multi-modal classification network obtain high performance in experiments and can be used to assist radiologists in more easily localizing and classification of prostate cancer.

## Data Availability

The prostate MR images data sets generated and analyzed in this article were obtained from the PROSTATEx challenge held by Grand Challenges in conjunction with the SPIE Medical Imaging Symposium (https://PROSTATEx.grand-challenge.org/).
