# Supplementary material for: Computer-aided diagnosis of prostate cancer based on deep neural networks from multi-parametric magnetic resonance imaging
Source: Front Physiol. 2022 Aug 29;13:918381. doi: 10.3389/fphys.2022.918381 (PMC9465082; doi:10.3389/fphys.2022.918381)
Supplement: Supplementary file 1 [file Table1.DOCX]

Table S1 Performance of selected different optimizers, learning rate, Epoch and Batch size in the training network

| Batch size | Learning rate | Epochs | ADAM(%) | AdaGrad(%) | RMSProp(%) |
| --- | --- | --- | --- | --- | --- |
| 4 | 1e^-4^ | 50 | 65.23 | 72.73 | 56.30 |
|  |  | 100 | 56.49 | 76.38 | 54.81 |
|  |  | 150 | 52.57 | 82.55 | 63.92 |
|  |  | 200 | 62.65 | 76.84 | 50.45 |
|  | 1e^-5^ | 50 | 71.52 | 70.65 | 81.16 |
|  |  | 100 | 84.26 | 75.24 | 82.40 |
|  |  | 150 | 87.04 | 78.03 | 85.55 |
|  |  | 200 | 91.42 | 79.42 | 89.47 |
| 8 | 1e^-5^ | 50 | 79.47 | 61.79 | 79.62 |
|  |  | 100 | 82.46 | 68.13 | 82.74 |
|  |  | 150 | 86.39 | 71.35 | 82.31 |
|  |  | 200 | 89.81 | 63.62 | 86.56 |
