# Supplementary figures and images for: Computer-aided diagnosis of prostate cancer based on deep neural networks from multi-parametric magnetic resonance imaging
Source: Front Physiol. 2022 Aug 29;13:918381. doi: 10.3389/fphys.2022.918381 (PMC9465082; doi:10.3389/fphys.2022.918381)

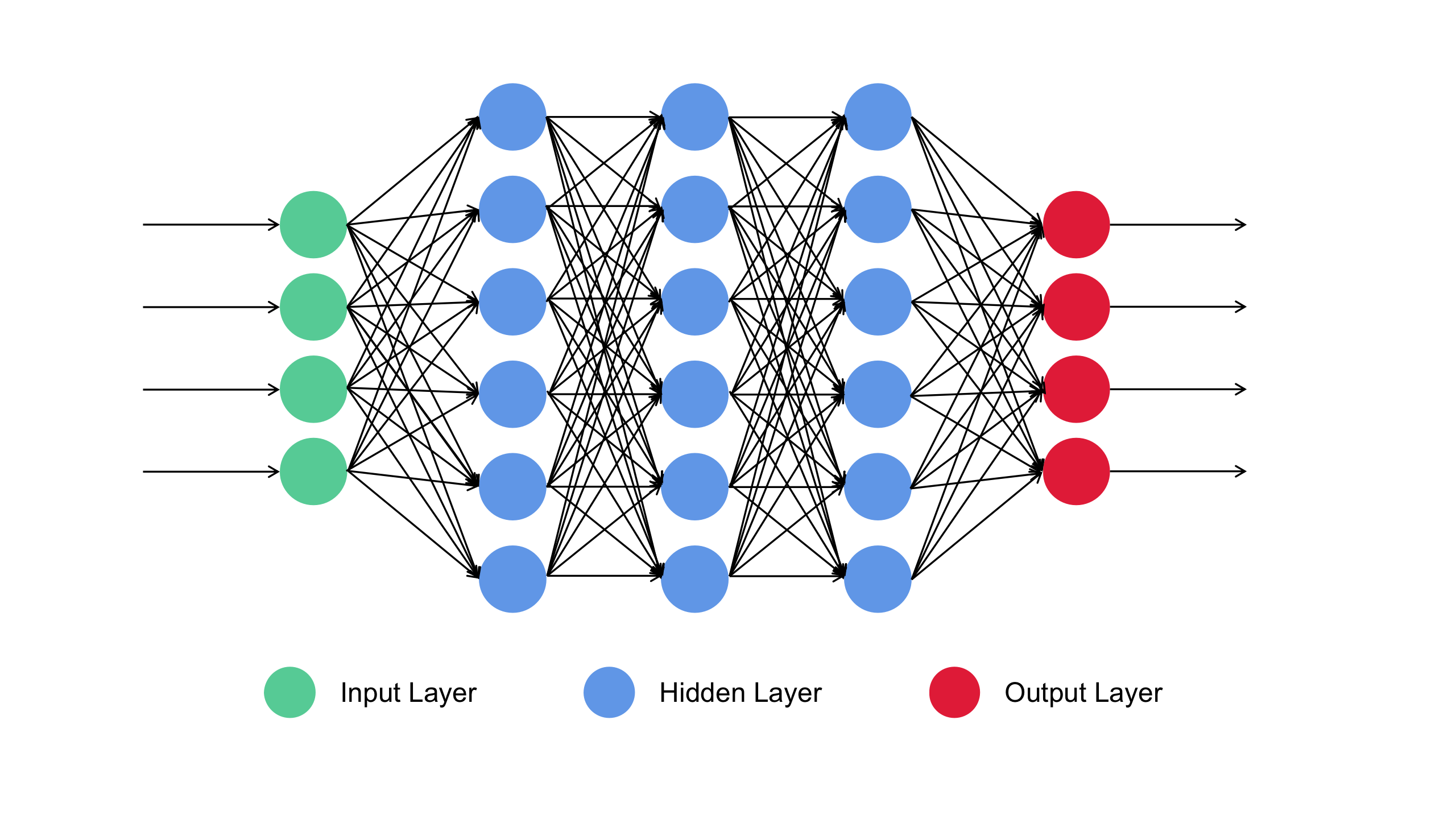

Supplement: Supplementary file 2 [file Image1.TIF]
