# Supplementary material for: Computer-aided diagnosis of prostate cancer based on deep neural networks from multi-parametric magnetic resonance imaging
Source: Front Physiol. 2022 Aug 29;13:918381. doi: 10.3389/fphys.2022.918381 (PMC9465082; doi:10.3389/fphys.2022.918381)
Supplement: Supplementary file 4 [file Table3.DOCX]

Table S3. Indicators of input tensor multi-modal classification network and five single-modal classification networks.

| **Modality** | **TPR** | **TNR** | **F1-score** | **AUC** | **Accuracy** |
| --- | --- | --- | --- | --- | --- |
| Input Tensor Multi-modal Classification Network | **0.90** | 0.82 | **0.8654** | **0.900** | **0.86** |
| Ktrans | **0.90** | 0.80 | 0.8571 | 0.853 | 0.85 |
| ADC | 0.89 | 0.72 | 0.8203 | 0.826 | 0.805 |
| T2-Weighted COR | 0.85 | 0.68 | 0.7834 | 0.741 | 0.765 |
| T2-Weighted SAG | 0.64 | **0.84** | 0.7636 | 0.735 | 0.74 |
| T2-Weighted TRA | 0.80 | 0.69 | 0.7583 | 0.775 | 0.745 |

ADC, apparent diffusion coefficient; AUC, area under curve; COR, coronal; TNR, true negative rate; TPR, true positive rate; SAG, sagittal; TRA, transverse.
