# Supplementary material for: Computer-aided diagnosis of prostate cancer based on deep neural networks from multi-parametric magnetic resonance imaging
Source: Front Physiol. 2022 Aug 29;13:918381. doi: 10.3389/fphys.2022.918381 (PMC9465082; doi:10.3389/fphys.2022.918381)
Supplement: Supplementary file 5 [file Table4.DOCX]

Table S4. Effect of the number of training samples on the model performance results.

| Sample size | **TPR** | **TNR** | **F1-score** | **AUC** | **Accuracy** |
| --- | --- | --- | --- | --- | --- |
| 50 | 0.72 | 0.64 | 0.6963 | 0.704 | 0.683 |
| 100 | 0.85 | 0.73 | 0.8102 | 0.823 | 0.796 |
| 150 | 0.92 | 0.76 | 0.8675 | 0.879 | 0.858 |
| 200 | 0.94 | 0.81 | 0.8854 | 0.908 | 0.883 |
| All | 0.95 | 0.82 | 0.8920 | 0.912 | 0.885 |

AUC, area under curve; TNR, true negative rate; TPR, true positive rate.
